# Alaptide from synchrotron powder diffraction data

**DOI:** 10.1107/S1600536810007750

**Published:** 2010-03-13

**Authors:** Jan Rohlíček, Jaroslav Maixner, Richard Pažout, Michal Hušák, Jana Cibulková, Bohumil Kratochvíl

**Affiliations:** a Department of Solid State Chemistry, Prague Institute of Chemical Technology, Technická 5, 166 28 Prague 6, Czech Republic; b Central Laboratories, Prague Institute of Chemical Technology, Technická 5, 166 28 Prague 6, Czech Republic

## Abstract

The title compound [systematic name: (8*S*)-8-methyl-6,9-diaza­spiro­[4.5]decane-7,10-dione], C_9_H_14_N_2_O_2_, consists of two connected rings, *viz.* a piperazine-2,5-dione (DKP) ring and a five-membered ring. The DKP ring adopts a slight boat conformation and the bonded methyl group is in an equatorial position. The five-membered ring is in an envelope conformation. In the crystal structure, inter­molecular N—H⋯O hydrogen bonds link mol­ecules into chains running parallel to the *c* axis.

## Related literature

For background to alaptide and its biological activity, see: Kasafírek *et al.* (1992 ▶); Hliňák *et al.* (1996 ▶). For a related structure, see: Symerský *et al.* (1987 ▶). For the original powder diffraction data, see: Maixner *et al.* (2009 ▶). For the synthetic procedure, see: Sturc & Kacafirek (1992 ▶). For a description of the Cambridge Structural Database, see: Allen (2002 ▶). For the March–Dollase orientation correction, see: (Dollase, 1986 ▶).
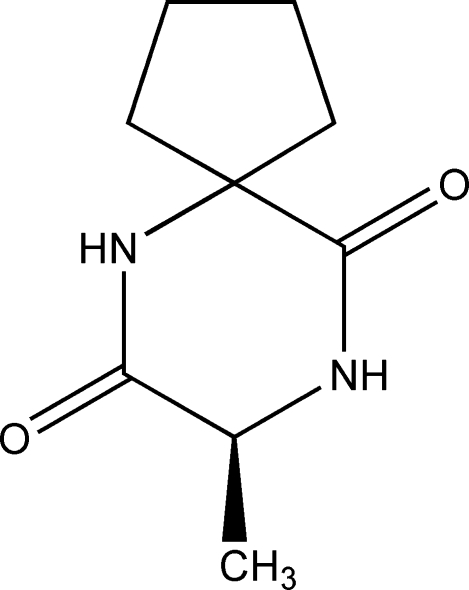


## Experimental

### 

#### Crystal data

C_9_H_14_N_2_O_2_
*M* *_r_* = 182.22
Orthorhombic, 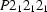

*a* = 21.14118 (7) Å
*b* = 7.22207 (2) Å
*c* = 6.14610 (3) Å
*V* = 938.41 (1) Å^3^
*Z* = 4
Synchrotron radiation, λ = 0.79984 Å
*T* = 293 K
Cylinder, 40 × 1 mm

#### Data collection

ID31 ESRF Grenoble diffractometer
Specimen mounting: capilary
Data collection mode: transmission
Scan method: step
2θ_min_ = 1.00°, 2θ_max_ = 48.01°, 2θ_step_ = 0.003°

#### Refinement

*R*_p_ = 0.058
*R*_wp_ = 0.089
*R*_exp_ = 0.023
*R*_Bragg_ = 0.102
χ^2^ = 15.210
15671 data points
53 parameters
37 restraints
H-atom parameters not refined

### 

Data collection: *ESRF SPEC* (Certified Scientific Software, 2003 ▶); cell refinement: *EXPO2004* (Altomare *et al.*, 1999 ▶); data reduction: *CRYSFIRE2004* (Shirley, 2000 ▶); program(s) used to solve structure: *EXPO2004*; program(s) used to refine structure: *GSAS* (Larson & Von Dreele, 1994 ▶); molecular graphics: *Mercury* (Macrae *et al.*, 2006 ▶) and *PLATON* (Spek, 2009 ▶); software used to prepare material for publication: *enCIFer* (Allen *et al.*, 2004 ▶).

## Supplementary Material

Crystal structure: contains datablocks global, I. DOI: 10.1107/S1600536810007750/lh2977sup1.cif

Rietveld powder data: contains datablocks I. DOI: 10.1107/S1600536810007750/lh2977Isup2.rtv

Structure factors: contains datablocks I. DOI: 10.1107/S1600536810007750/lh2977Isup3.hkl

Additional supplementary materials:  crystallographic information; 3D view; checkCIF report

## Figures and Tables

**Table 1:** Hydrogen-bond geometry (Å, °)

*D*—H⋯*A*	*D*—H	H⋯*A*	*D*⋯*A*	*D*—H⋯*A*
N4—H41⋯O8^i^	0.86	2.10	2.929 (3)	164
N7—H71⋯O13^ii^	0.86	2.01	2.826 (3)	159

Symmetry codes: (i) 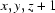; (ii) 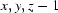.

**Fig. 1.**
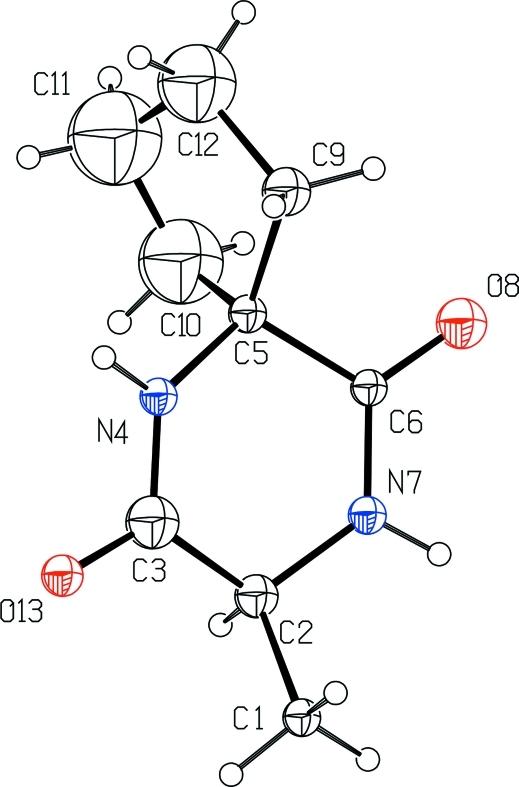
The molecular structure of alaptide showing the atomic numbering. Displacement spheres are drawn at 30% probability level.

**Fig. 2.**
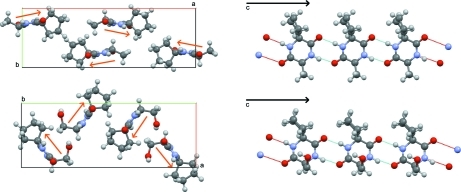
Comparison of molecular packing (left - arrows show directions of dipeptide rings) and hydrogen bonding system (right) of two structures. Top: Structure of alaptide, bottom: Structure of (8S)-8-Hydroxymethyl-6,9-diazaspiro[4.5]decane-7,10-dione.

**Fig. 3.**
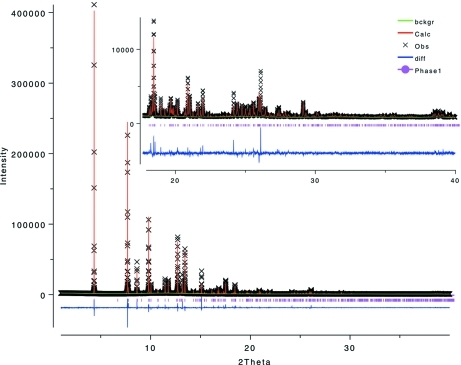
The final Rietveld plot showing the measured data (black thin-plus), calculated data (red line) and difference curve (blue line). Calculated positions of the reflection are shown by vertical bars.

## supplementary crystallographic information

### Comment

Alaptide is a small molecule belonging to the group of spirocyclic dipeptides (Kasafírek *et al.*, 1992). The systematic research during the last twenty years has shown a positive effect of alaptide and its derivatives on the memory of animals and on healing of burns (Hliňák *et al.*, 1996).

The molecular structure of the title compound is shown in Fig. 1. The crystal structure contains two types of intermolecular N—H···O hydrogen bonds between DKP rings. The DKP ring adopts a slight boat conformation and is connected via the spiro junction to a five-membered carbon ring which is in an envelope conformation. The methyl group bonded to the dipeptide ring is in an equatorial position. A search in the Cambridge Structural Database (Allen, 2002) found the crystal structure of a similar type of molecule, namely: (8*S*)-8-Hydroxymethyl-6,9-diazaspiro[4.5]decane-7,10-dione (CSD refcode FEPFOV; Symerský *et al.*, 1987). This structure has the same spacegroup and comparable unit-cell parameters as the reported structure of the title copmound. Two similar hydrogen bonds N—H···O connecting DKP rings of neighboring molecules occur in both crystal structures. In both structures, the hydrogen bonding connects molecules to form one-dimensional chains. The third hydrogen bond O—H···O is missing in the structure of alaptide, which causes a different formation of extended chains in these structures, see Fig. 2.

### Experimental

The title compound was synthesized according to the procedure of Sturc & Kacafirek (1992). Alaptide was crystallized from various solvents in order to check polymorphism, but only one solid form was found (Maixner *et al.*, 2009). The sample for measurement was recrystallized from methanol by slow evaporation technique.

### Refinement

X-Ray diffraction data were collected on the high resolution diffractometer ID31 of the European Synchrotron Radiation Facility. The monochromatic wavelength was fixed at 0.79984 (4) Å. Si (111) crystal multi-analyzer combined with Si (111) monochromator was used (beam offset angle α = 2°). A rotating 1-mm-diameter borosilicate glass capillary with alaptide powder was used for the experiment. Data were measured from 1.002° 2θ to 48.012° 2θ at the room temperature, steps scans were set to 0.003° 2θ.

Indexation was done in CRYSFIRE 2004 (Shirley, 2000) package. It confirmed previously presented unit-cell parameters and space group (Maixner *et al.*, 2009): *a* = 21.136 (4), *b* = 7.212 (4), *c *= 6.126 (3) Å, *P*2_1_2_1_2_1_, V = 933.8 (8) Å^3^, and *Z* = 4. The structure was solved by using direct space methods implemented in EXPO2004 package (Altomare *et al.*,1999). All non-hydrogen atoms were found in the structure solution process. Hydrogen atoms were placed in their theoretical positions and structure was refined by Rietveld method as implemented in *GSAS* (Larson & Von Dreele, 1994). Bonds, angles and planar group restraints were used during refinement. At final stages atomic coordinates and *U*_iso_ parameters of non-hydrogen atoms were refined to the final agreement factors *R*_p_ = 0.059 and *R*_wp_= 0.089. The diffraction profiles and differences between the measured and calculated profiles are shown in Fig. 3.

The isotropic displacement parameters of atoms C10, C11 and C12 are large compared to those of the other atoms. A disorder model was attempted but this did not improve the refinement and therefore was not used.

### Crystal data

**Table:**

C_9_H_14_N_2_O_2_	*F*(000) = 392
*M**_r_* = 182.22	*D*_x_ = 1.290 Mg m^−^^3^
Orthorhombic, *P*2_1_2_1_2_1_	Synchrotron radiation, λ = 0.79984 Å
Hall symbol: P 2ac 2ab	*T* = 293 K
*a* = 21.14118 (7) Å	Particle morphology: no specific habit
*b* = 7.22207 (2) Å	white
*c* = 6.14610 (3) Å	cylinder, 40 × 1 mm
*V* = 938.41 (1) Å^3^	Specimen preparation: Prepared at 293 K and 101 kPa
*Z* = 4	

### Data collection

**Table:**

ID31 ESRF Grenoble diffractometer	Data collection mode: transmission
Radiation source: synchrotron	Scan method: step
Si(111)	2θ_min_ = 1.00°, 2θ_max_ = 48.01°, 2θ_step_ = 0.003°
Specimen mounting: capilary	

### Refinement

**Table:**

Least-squares matrix: full	53 parameters
*R*_p_ = 0.058	37 restraints
*R*_wp_ = 0.089	0 constraints
*R*_exp_ = 0.023	H-atom parameters not refined
*R*_Bragg_ = 0.102	Weighting scheme based on measured s.u.'s *w* = 1/σ(Y_obs_)^2^
χ^2^ = 15.210	(Δ/σ)_max_ = 0.06
15671 data points	Background function: Shifted Chebyschev
Excluded region(s): no	Preferred orientation correction: March–Dollase (Dollase, 1986); direction of preferred orientation is 101; MD = 0.93

### Fractional atomic coordinates and isotropic or equivalent isotropic displacement parameters (Å^2^)

**Table:**

	*x*	*y*	*z*	*U*_iso_*/*U*_eq_	
C1	−0.08373 (10)	0.2121 (8)	−0.0450 (5)	0.027 (3)*	
C2	−0.01869 (8)	0.3035 (3)	−0.0158 (4)	0.035 (3)*	
C3	0.01033 (9)	0.27000 (17)	0.2032 (3)	0.052 (3)*	
N4	0.07052 (9)	0.2345 (4)	0.2137 (3)	0.026 (2)*	
C5	0.11718 (7)	0.2470 (3)	0.0365 (3)	0.027 (3)*	
C6	0.08529 (8)	0.23092 (17)	−0.1846 (3)	0.025 (3)*	
N7	0.02321 (9)	0.2505 (4)	−0.1936 (3)	0.027 (2)*	
O8	0.11977 (11)	0.2014 (4)	−0.3425 (4)	0.047 (2)*	
C9	0.16843 (14)	0.0925 (6)	0.0590 (5)	0.043 (4)*	
C10	0.15361 (16)	0.4304 (5)	0.0487 (5)	0.133 (6)*	
C11	0.20873 (16)	0.3907 (9)	0.1995 (5)	0.165 (5)*	
C12	0.22476 (14)	0.1870 (9)	0.1704 (8)	0.114 (4)*	
O13	−0.02052 (12)	0.2734 (4)	0.3727 (4)	0.0319 (18)*	
H11	−0.0992	0.2386	−0.1875	0.0346*	
H12	−0.1121	0.2595	0.0598	0.0346*	
H13	−0.0795	0.0821	−0.0282	0.0346*	
H21	−0.025	0.4337	−0.0286	0.0516*	
H91	0.181	0.0494	−0.0796	0.063*	
H92	0.1531	−0.0061	0.1445	0.063*	
H101	0.1686	0.4665	−0.0916	0.18*	
H102	0.1276	0.5269	0.1053	0.18*	
H111	0.2441	0.4664	0.1615	0.2445*	
H112	0.197	0.4181	0.3464	0.2445*	
H121	0.2617	0.1746	0.089	0.168*	
H122	0.2309	0.1335	0.313	0.168*	
H71	0.006	0.2305	−0.3181	0.0296*	
H41	0.0848	0.2013	0.3384	0.0271*	

### Geometric parameters (Å, °)

**Table:**

O8—C6	1.232 (3)	N4—H41	0.86
O13—C3	1.229 (3)	N7—H71	0.86
N4—C3	1.300 (3)	C1—H11	0.95
N4—C5	1.472 (3)	C1—H12	0.94
N7—C2	1.458 (3)	C1—H13	0.95
N7—C6	1.321 (3)	C2—H21	0.95
C1—C2	1.536 (4)	C9—H91	0.95
C2—C3	1.499 (3)	C9—H92	0.94
C5—C6	1.521 (3)	C10—H101	0.95
C5—C9	1.561 (4)	C10—H102	0.95
C5—C10	1.534 (4)	C11—H111	0.96
C9—C12	1.534 (5)	C11—H112	0.96
C10—C11	1.516 (5)	C12—H121	0.93
C11—C12	1.520 (9)	C12—H122	0.97
			
C3—N4—C5	127.39 (18)	C2—C1—H13	109
C2—N7—C6	126.85 (18)	H11—C1—H12	110
N7—C2—C1	110.1 (2)	H11—C1—H13	109
N7—C2—C3	112.48 (16)	H12—C1—H13	110
C1—C2—C3	113.7 (2)	N7—C2—H21	106
O13—C3—N4	118.8 (2)	C1—C2—H21	107
O13—C3—C2	122.7 (2)	C3—C2—H21	107
N4—C3—C2	118.50 (17)	C5—C9—H91	111
N4—C5—C6	111.05 (14)	C5—C9—H92	111
N4—C5—C9	110.8 (2)	C12—C9—H91	109
N4—C5—C10	110.7 (2)	C12—C9—H92	111
C6—C5—C9	109.39 (18)	H91—C9—H92	111
C6—C5—C10	109.39 (18)	C5—C10—H101	111
C9—C5—C10	105.3 (2)	C5—C10—H102	111
O8—C6—N7	124.94 (19)	C11—C10—H101	110
O8—C6—C5	117.03 (17)	C11—C10—H102	111
N7—C6—C5	118.02 (16)	H101—C10—H102	109
C5—C9—C12	105.1 (3)	C10—C11—H111	110
C5—C10—C11	104.6 (3)	C10—C11—H112	110
C10—C11—C12	106.4 (4)	C12—C11—H111	111
C9—C12—C11	108.1 (3)	C12—C11—H112	112
C3—N4—H41	116	H111—C11—H112	108
C5—N4—H41	116	C9—C12—H121	112
C2—N7—H71	117	C9—C12—H122	109
C6—N7—H71	116	C11—C12—H121	110
C2—C1—H11	109	C11—C12—H122	108
C2—C1—H12	109	H121—C12—H122	110

### Hydrogen-bond geometry (Å, °)

**Table:**

*D*—H···*A*	*D*—H	H···*A*	*D*···*A*	*D*—H···*A*
N4—H41···O8^i^	0.86	2.10	2.929 (3)	164
N7—H71···O13^ii^	0.86	2.01	2.826 (3)	159

Symmetry codes: (i) *x*, *y*, *z*+1; (ii) *x*, *y*, *z*−1.
